# Bovine Immune Factors Underlying Tick Resistance: Integration and Future Directions

**DOI:** 10.3389/fcimb.2017.00522

**Published:** 2017-12-19

**Authors:** Luïse Robbertse, Sabine A. Richards, Christine Maritz-Olivier

**Affiliations:** Department of Genetics, Faculty of Natural and Agricultural Sciences, University of Pretoria, Pretoria, South Africa

**Keywords:** cattle, tick, resistance, tick resistance, immune factors, parasite, host

## Abstract

The mechanisms underlying tick resistance within and between cattle breeds have been studied for decades. Several previous papers on bovine immune parameters contributing to tick resistance discussed findings across DNA, RNA, protein, cellular, and tissue levels. However, the differences between bovine host species, tick species and the experimental layouts were not always taken into account. This review aims to (a) give a comprehensive summary of studies investigating immune marker differences between cattle breeds with varying degrees of tick resistance, and (b) to integrate key findings and suggest hypotheses on likely immune-regulated pathways driving resistance. Experimental issues, which may have skewed conclusions, are highlighted. In future, improved experimental strategies will enable more focused studies to identify and integrate immune markers and/or pathways. Most conclusive thus far is the involvement of histamine, granulocytes and their associated pathways in the tick-resistance mechanism. Interestingly, different immune markers might be involved in the mechanisms within a single host breed in contrast to between breeds. Also, differences are evident at each tick life stage, limiting the level to which datasets can be compared. Future studies to further elucidate immune molecule dynamics across the entire tick life cycle and in-depth investigation of promising markers and pathways on both molecular and cellular level are in dire need to obtain a scientifically sound hypothesis on the drivers of tick resistance.

## Introduction

The economic importance of ticks and the need to control them was realized alongside the discovery of their potential as vectors of harmful parasites, particularly to livestock (Hunter and Hooker, 1907; Theiler, 1911). The variability in the degree to which cattle display resistance to ixodid ticks was first suggested by Johnston and Bancroft (1918). It is known that tick resistance in cattle varies from more tick-susceptible *Bos taurus taurus* (*B. t. taurus*) to more tick-resistant *B. t. indicus* breeds, between bovine crosses as well as within a single cattle breed (George et al., 1985; Rechav et al., 1991b; Mattioli and Cassama, 1995; Mwangi et al., 1998; Mattioli et al., 2000; Nascimento et al., 2011). However, the biological factors underlying bovine resistance to tick infestation are still poorly understood. Tick resistance is a multi-factorial trait suggested to involve host-related factors such as sex, age, lactation, grooming behavior, skin composition and host surface area, coat length and environmental factors (Wharton et al., 1970; Seifert, 1971; Doube and Wharton, 1980; Binta and Cunningham, 1984; Ali and de Castro, 1993; Meltzer, 1996; Norval et al., 1996; Mattioli, 1998; Martinez et al., 2006; Kongsuwan et al., 2010). It is also well established that the tick-resistance phenotype is heritable, as is evident from breed-specific resistance patterns. Furthermore, it was proposed that tick attachment sites on resistant cattle rapidly become unsuitable for feeding due to host immune responses (Roberts, 1968b). The majority of studies indicate that resistance is acquired through exposure to ticks (Wagland, 1975, 1980; George et al., 1985; Momin et al., 1991) and that resistance is acquired sooner and to a higher degree in *B. t. indicus* than in *B. t. taurus* breeds (Riek, 1962; Wagland, 1978, 1980; Rechav et al., 1990). This phenotype only becomes apparent after subsequent (and not initial) tick exposure in *B. t. taurus, B. t. indicus* and mixed breed cattle (Roberts, 1968a; Wagland, 1975; Hewetson and Lewis, 1976).

To further elucidate potential mechanisms underlying differences in tick resistance, several studies have investigated host immune responses toward ticks on a cellular and molecular level. Gaining an understanding of the molecular mechanisms underlying tick-resistance mechanism will be advantageous in the identification of specific genomic alterations or specific markers that could lead to the ability to screen cattle for their potential resistance status without prolonged tick-infestation trials or counts. This would be helpful in breeding more tick-resistant cattle. In this regard, it was shown that although host-resistance to tick infestation and product yield does not correlated in Holstein-Friesian cattle (Jonsson et al., 2000), but carriers of both *B. t. taurus* and *B. t. indicus* genes may suffer from a trade-off between animal-derived product yield and tick resistance (Wang et al., 2007). A clear understanding of tick-resistance mechanisms would also be beneficial to vaccine trials, where a difference in the resistance status of individual animals could skew results and therefore make accurate data interpretation more difficult. Furthermore, more effective vaccine formulations could be devised as vaccine efficacy is hindered by the modulation of host immune responses through tick saliva (Kazimírová and Štibrániová, 2013). Knowledge regarding molecular mechanisms underlying tick resistance could allow for the optimal selection of appropriate adjuvant/vaccine formulation strategies to provide a cross-breed protective response.

This review therefore provides a summary of studies performed up to date in cattle blood and skin tissue, with critical evaluation of findings followed by hypotheses on key role players, possible immune-regulated pathways as well as improvements for consideration when planning future experiments. Several recent studies were published pertaining to genetic associations with regards to the tick-resistance phenotype (Mota et al., 2016a,b, 2017; Junqueira et al., 2017; Sollero et al., 2017), however, these are outside the scope of this summary. This review should provide readers with the basic knowledge and a critical evaluation of findings to date to make informed decisions for future studies investigating the tick-host-interface with a focus on resistance. Due to differences in experimental layouts, which might skew data interpretation and comparisons such as tick life stage, tick species and type of bovine comparison (between or within breeds), are provided in Supplementary Table 1.

## Blood

Immune cells originate from hematopoietic stem cells in the bone marrow with naïve and mature forms of these cells circulating in the blood and lymphatic systems. Here, they encounter foreign molecules that lead to their proliferation, differentiation and maturation (Janeway et al., 2001). However, due to the constant circulation and changing dynamics of immune response components, experimental designs (especially time points) must be chosen carefully. Various gene expression, translational and cytological studies have investigated blood to elucidate immune responses linked to tick resistance/susceptibility in cattle and these are described in the next section.

### Gene expression studies in the blood of tick-infested cattle

Gene expression studies of peripheral blood mononuclear cells identified transcripts for IL-2, IL2Rα, TNFα, and CCR1 to be significantly upregulated in resistant cattle relative to susceptible cattle, while a significantly higher expression of *CXCL10* was detected in susceptible Holstein-Friesian compared to resistant Brahman cattle (Piper et al., 2009). Pathway analysis indicated that genes that are more highly expressed in the resistant breed are associated with the hematopoietic cell lineage and cytokine-cytokine receptor interaction pathways. Another study found a significant upregulation of *CD25, IL10, FoxP3*, and *CXCL10* in samples from cattle infested with larvae compared to samples obtained from uninfested animals. In susceptible animals, *CXCL8* was downregulated in susceptible animals 24 and 48 h after infestation compared to samples from uninfested animals (Domingues *et al*., 2014). Although *CXCL10* was identified in both studies as differentially regulated, major differences in the study designs hindering any direct comparisons. Piper et al. (2009) obtained blood samples at the peak of tick infestation without reference to a specific time point after infestation and found significantly higher chemokine expression in susceptible compared to resistant cattle breeds. Domingues et al. (2014) on the other hand compared tick-infested versus tick-uninfested cattle of the same breed and identified an increase in *CXCL10* in resistant animals 48 h and an increase in susceptible animals 24 h after tick infestation. Therefore, the role of CXCL10 remains to be confirmed in future studies and its contribution to resistance pathways elucidated.

### Translational studies in the blood of tick-infested cattle

#### Immunoglobulins

A link between tick resistance and immunoglobulins was proposed in 1987 by Rechav and colleagues who found a positive correlation between tick numbers and total serum gamma globulin levels in naturally infested *B. t. taurus* and *B. t. indicus* cattle. During the acquisition of tick resistance, a negative correlation between tick weight and total serum gamma globulin levels was, however, documented in *B. t. taurus* cattle infested with *Rhipicephalus decoloratus* (Rechav et al., 1991a). This discrepancy could be a result of differences in experimental design, as the animals studied by (Rechav et al., 1991a) were most likely in the process of acquiring tick resistance as opposed to the more established resistance of cattle studied by Rechav (1987). In this regard, resistance in the former study was supported by reduced tick weights only.

In general, the number of ticks feeding on cattle were found to positively correlate with salivary gland specific IgG levels in previously infested (Sahibi et al., 1998) and naïve (Cruz et al., 2008) *B. t. taurus* cattle. Cruz et al. (2008) furthermore reported that there was no change in the avidity of antibodies developed by either *Rhipicephalus microplus* resistant or susceptible animals against salivary soluble extracts (Cruz et al., 2008).

Differences in the IgG1 isotype was observed when comparing cattle breeds displaying varying tick-resistance phenotypes. After multiple infestations of tick-naïve cattle, IgG1 levels (against several tick extracts) were found to be significantly higher in susceptible compared to resistant cattle (Piper et al., 2017), with similar results obtained the studies of Garcia et al. (2017) and Piper et al. (2009). Although no differences were observed for tick-naïve animals at the beginning of the study by Piper et al. (2017), higher tick-saliva specific IgG1 levels were seen before and at the beginning of the first infestation in the tick-resistant cattle breed by Garcia et al. (2017). Yet, Kashino et al. (2005) reported a decrease of IgG1 upon heavy tick infestation in naturally infested susceptible animals, compared to resistant animals (Kashino et al., 2005). As such, the question arises whether studies done under controlled housing conditions and those done under field conditions with natural infestation, and possible co-infections/infestations, can be compared.

In contrast to IgG1 levels, no significant differences were identified for the IgG2 isotype between breeds (Piper et al., 2009, 2017), with similar results seen by Garcia et al. (2017) for resistant animals throughout the study. The latter study does however describe an increase of this isotype in the susceptible breed at the third infestation compared to the baseline. At the same time point, IgG2 levels were significantly higher in susceptible compared to resistant animals. Again, in contrast, Kashino et al. (2005) identified decreased levels of IgG2 in naturally infested susceptible compared to resistant animals during heavy infestation.

Only two studies have investigated IgE levels between resistant and susceptible cattle breeds. Garcia et al. (2017) determined levels of total IgE in the sera of cattle infested with *R. microplus*. No difference in these levels were noted between resistant and susceptible breeds. Tick-specific IgE antibody levels, however, were shown to be significantly lower in resistant compared to susceptible animals during heavy infestation as well as during subsequent light infestation (Kashino et al., 2005). This difference in IgE levels between cattle breeds seems to be a result of an increase in this immunoglobulin in susceptible animals instead of a decrease in resistant animals. The same trend was seen in some studies investigating IgG isotypes. IgE with associated receptors and cellular responses are believed to have evolved to counter helminths and other parasites that cannot be phagocytosed (Fitzsimmons et al., 2014). In this paper, we propose a role for IgE-dependent responses as one of the drivers of resistance (see sections Dynamics of Granulocytes and Histamine and their Suggested Involvement in the Tick- Resistance Mechanism Over the Tick Lifecycle and Future Directions: Potential Drivers Involved in Tick Resistance), and as such, daily data on the IgE levels throughout the period of tick attachment and subsequent life stages will be of great importance.

Considering the consensus from the majority of studies, resistant animals seem to display a more constant tick-specific immunoglobulin isotype profile with fewer changes observed throughout infestation cycles. Susceptible animals on the other hand show an increase of tick-specific immunoglobulin levels over multiple infestations. Differences in host immune responses are furthermore evident by the observation that a great variation in tick salivary gland extract profiles are recognized between individual cattle sera (Cruz et al., 2008). Furthermore, sera from a resistant compared to a susceptible cattle breed reacted with more tick salivary proteins, which require further investigation (Garcia et al., 2017). On the other hand, differences in tick numbers and thus the amount of tick antigens in different hosts also requires more in-depth studies to determine immune responses independent of varying tick numbers.

#### Other

Additional host immune components have also been associated with tick resistance to date, including histamine, complement, acute-phase proteins and bovine lymphocyte antigens. Increased histamine and complement levels were found to be associated with lower tick numbers and resistant animals, respectively (Riek, 1962; Wambura et al., 1998; Zhao et al., 2013). Three proteins of the acute-phase response, which is generally initiated in response to tissue damage (Baumann and Gauldie, 1994), were linked to the tick-resistance mechanism by Carvalho et al. (2008). Briefly, susceptible Holstein-Friesian cattle showed a significant increase in haptoglobulin levels resulting from heavy tick infestation as well as constantly higher alpha-1 acid glycoprotein levels compared to resistant Nelore (*B. t. indicus*) animals. This might be a result of increased tick numbers and the associated increase in tissue damage on susceptible animals. Lastly, only during intense infestation did the more resistant cattle breed show higher levels of serum amyloid A when compared to the more susceptible cattle breed. In two separate studies, a total of 19 bovine lymphocyte antigens were tested in blood collected from a mixed breed cattle population with no overlapping findings. In total, two antigens were significantly associated with tick resistance (W8 and W16) and three with tick-susceptibility (W5, W6, CA31) (Stear et al., 1984, 1989). Additional data is therefore required to resolve knowledge gaps in the pathways associated with the above-mentioned compounds.

### Cytological studies

#### Identification of immune cell subtypes in circulating blood of tick-infested cattle using associated markers

Identification and quantification of immune cell subtypes in circulating blood has been performed in *B. t. indicus, B. t. taurus* and mixed breed cattle with the use of associated markers. Piper et al. (2017) did not find any significant differences while Piper et al. (2009) identified significantly higher levels of CD4+, CD25+ activated and WC1+ γδ T-cell populations in more tick-resistant cattle. Significantly higher levels of CD14+ monocyte and MHC II presenting cells were obtained in more tick-susceptible cattle. As these cell subtypes are known to be associated with a variety of immune responses and pathways, linking them to a putative resistance mechanism will only be possible when analyzing them in combination with additional markers (**Figure 2**).

#### Identification of immune cell subtypes in circulating blood of tick-infested cattle using morphological characteristics

The cellular composition of blood is regarded as an important identifier of the overall health of humans and animals and as such changes in the percentage of different white blood cell populations may be used as an indication of a systemic immune response. Three main studies have relied on the use of different white blood cell population counts (basophils, eosinophils, lymphocytes, total leukocytes, neutrophils, monocytes) in describing the immune response of cattle with varying levels of tick resistance with no differential regulation identified to date (Brown et al., 1984; Rechav, 1987; Rechav et al., 1990). Only blood eosinophil levels were significantly higher in the more susceptible *B. t. taurus* breed (which carried more ticks) in the study by Rechav et al. (1990). The investigation of cell subtypes in blood represents a daunting task as shown by the lack of identified differential regulation of markers obtained. In contrast, research to date has detected significant differences between hosts with varying tick-resistance status in skin tissue (refer to skin section below). This is the case since the dynamics of immune cells in blood only provide a snapshot of what is occurring at a specific time point. Experimental layouts must thus be considered carefully as studies incorporating and comparing several time points and/or tissues might represent a more realistic view of immune drivers of resistance.

## Skin tissue

The skin represents the first site of encounter to tick infestation and thus the first line of host immune defense. Upon penetration and successful attachment, ixodid ticks alternate salivation and blood intake every 5–20 min (Francischetti et al., 2009). Numerous salivary components mediate suppression of host responses such as blood coagulation, immunity, inflammation and the ability of the host to develop new blood vessels (Hovius et al., 2008; Kazimírová and Štibrániová, 2013; Kotál et al., 2015). The identification of immunological defense responses at the site of tick infestation have been extensively studied, as evident from the next section.

### Gene expression studies in the skin of tick-infested cattle

Based on transcriptional studies in cattle skin, three studies have identified the involvement of the complement cascade in the feeding of ticks in both susceptible and resistant cattle (Wang et al., 2007; Piper et al., 2010; Carvalho et al., 2014). The complement system is composed of a number of molecules and plays a vital part in the immune system for the clearance of foreign cells via a number of mechanisms (Nesargikar et al., 2012). Upregulation of gene expression for complement components in tick-resistant cattle (*C1QA*) (Wang et al., 2007) and tick-susceptible cattle (*complement component 3*) (Piper et al., 2010) have been shown, while the general pathway downregulation of complement has also been described in susceptible animals (Carvalho et al., 2014). More in-depth studies are required regarding these components potentially involved in the tick-resistance mechanism. This is due to the various complement components investigated to date combined with differences amongst results reported. Interestingly, all immunoglobulin associated transcripts were identified to be more abundant in less resistant animals (Wang et al., 2007; Piper et al., 2010) which correlates with findings obtained from blood and could be linked to increased tick numbers on these animals. Furthermore, CD14 (on transcriptional level in skin and translational level in blood) was identified in both tissues to be associated with tick-susceptibility (Piper et al., 2008, 2009). CD14 is known to be a marker for monocytes and macrophages and can therefore be involved in several immune response mechanisms (Ziegler-Heitbrock and Ulevitch, 1993).

Other components that were found to be upregulated in susceptible cattle include transcripts for IL13RA1, CD44, CD63, TNFα, IL-1β, IL-10, NFKBp50, CD1a, CCR-1, CCL2, CCL26, TLR9, MyD88, CD14, FTH1, BDA20, and Traf-6 (Wang et al., 2007; Piper et al., 2008; Nascimento et al., 2011). However, no transcript was reported in more than one of these studies and as such all require validation. Most recently, Franzin et al. (2017) reported on a microarray study of skin from uninfested cattle, larvae (2 days after larvae infestation) and nymph (9 days after larvae infestation) life stages fed on *B. t. taurus* and *B. t. indicus* breeds. Samples were compared within and between breeds. An observed allergic contact-like dermatitis was found to be delayed in susceptible animals detected by the involvement of *IL-6, CXCL-8, CCL-2, HMGB1, ISG15*, and *PKR* which in turn result in the production of chemokines and cytokines involved in the inflammatory response. In another study, downregulation of inflammatory response gene expression was observed within 24 h after tick infestation in susceptible animals, while at the 48-h sampling point genes associated with antigen presentation and oxidative stress were found to be upregulated in resistant cattle (Carvalho et al., 2014). One study identified *CXCL-8* expression as being downregulated in resistant cattle between different genetic crossbred cattle groups from which skin and lymph node samples were obtained 9 days after larvae challenge (Regitano et al., 2008). However, it was unclear to which tissue this finding refers to. Differential gene expression was furthermore identified for genes encoding Blimp-1 (Kongsuwan et al., 2010), cathepsin L2 precursor, MHC class antigen I (Nascimento et al., 2011), various adhesion molecules (Carvalho et al., 2010), TNF receptor-associated factor 6, TATA-binding protein, lumican and beta-2 microglobulin (Marima, 2017).

Due to the variation in experimental designs of different studies, caution should be taken when trying to compare results emanating from transcriptional studies, especially with regards to sampling time points. Since gene expression rarely involves absolute quantification and is based on the relative quantification of transcripts between two populations or between transcripts and reference genes under a specific set of conditions, the results generated may be study specific. RNA sequencing would be an alternative approach not yet utilized in this field of study for the obtainment of large-scale results based on absolute quantification which could allow the identification of novel transcripts. In addition to this, studies on a protein and cellular level, to validate potentially relevant findings from gene expression studies, should be undertaken.

### Translational studies and metabolites in the skin of tick-infested cattle

Few studies investigated immune factors underlying the tick-resistance mechanism in skin of cattle on the protein or metabolite level. To date, only one paper has focused on proteins. However, no significant findings regarding proteins directly involved in immune response pathways were identified (Kongsuwan et al., 2010). On a metabolite level, higher histamine levels were linked to tick resistance by Schleger et al. (1981) and Willadsen et al. (1979). These findings also correlate with results obtained from studies done on blood (Riek, 1962; Zhao et al., 2013). Histamine is an immunomodulator produced by a variety of cell types including mast cells, basophils, dendritic cells, and T-cells and can regulate both innate as well as adaptive immune response cells (O'Mahony et al., 2011). The expression of the histidine decarboxylase, which results in the decarboxylation of L-histidine and subsequent production of histamine, is influenced by several immune factors including a variety of cytokines. This secondary metabolite regulates, amongst others, antigen-specific Th1 and Th2 cells in addition to antibody isotype responses (Jutel et al., 2006). Histamine seems to be an effector molecule in tick resistance. This is evident from studies showing that histamine injection at tick attachment sites lead to detachment of some tick larvae, indicating a direct involvement of histamine rather than a general inflammatory reaction being the cause of tick rejection (Kemp and Bourne, 1980).

Furthermore, higher tick numbers were observed in cattle treated with an antihistaminic drug (Tatchell and Bennett, 1969). A response localized to the sites of skin damage is generally the first immunological reaction of a body. Histamine is well documented to be involved in proinflammatory responses and the immediate-type hypersensitivity response, characterized by increased vascular permeability, smooth muscle contractions, activation of certain nerves, wheal-and-flare reactions and itch responses (O'Mahony et al., 2011). Acquired resistance was linked to the occurrence of a hypersensitivity reaction to tick salivary gland components (Riek, 1962). The type of hypersensitivity is, however, not known since contradicting results have been obtained (Kemp et al., 1986; Smith et al., 1989; Latif et al., 1991; Bechara et al., 2000; Piper et al., 2010; Prudencio et al., 2011; Marufu et al., 2013).

### Cytological studies

#### Identification of immune cell subtypes via surface markers at the site of tick attachment

On a cellular level, two potential cell subtypes have been identified across independent studies. Markers used for the identification of γδ T-lymphocytes were found to be present in higher levels in resistant compared to susceptible animals (Constantinoiu et al., 2010; Franzin et al., 2017). Gamma delta T-cells are suggested to function as regulatory T-cells in bovines (Hoek et al., 2009).

The expression of CD3+ T-lymphocytes was found to be increased at different time points in *B. t. indicus* cattle (compared to *B. t. taurus)* in two studies (Constantinoiu et al., 2010; Franzin et al., 2017). This could be explained by different infestation protocols. Constantinoiu et al. (2010) made use of naïve cattle which were infested weekly with *R. microplus* larvae. Samples were taken at 1 day, one, 3 and 7 weeks post-primary infestation. CD3+ T-lymphocytes were found to be more abundant in resistant cattle at 1 day and at 3 weeks. In contrast, Franzin et al. (2017) used naïve cattle, which were challenged only once. Samples were taken at 2 and 9 days post infestation and significantly higher CD3+ T-lymphocyte levels were found in resistant compared to susceptible animals for the later sampling timepoint only. Based on the above studies and the importance of CD3+ T-lymphocytes in innate and adaptive immune responses, these cells are likely involved in the tick-resistance mechanism.

#### Neutrophils at the site of tick attachment

Although an increase in neutrophil levels at the site of tick attachment is well described and has been related to the number of previous tick exposures (Allen et al., 1977; Binta and Cunningham, 1984; Brown et al., 1984; Gill, 1986; Walker and Fletcher, 1986), no differences have been reported from studies investigating the association of neutrophils to tick resistance. This led to the hypothesis that this cell type is not linked to the tick-resistance mechanism. Latif et al. (1991) found a decrease in the infiltration of neutrophils in less resistant (*B. t. indicus* and *B. t. taurus*) animals when compared to resistant Zebu cattle infested with *Rhipicephalus appendiculatus* nymphs. In contrast, no differences for cattle infested with *Amblyomma variegatum* nymphs (comparing resistant and more susceptible Zebu animals) were found. Furthermore, no differences in the number of neutrophils at the site of adult *R. microplus* attachment could be observed between resistant and susceptible breeds by Carvalho et al. (2010). The same results were obtained by Marufu et al. (2014) for naturally infested cattle. It is evident that there is an increase in the number of neutrophils upon larvae and adult tick infestation, where equal numbers of this cell subtype were found to be present irrespective of the resistance classification of the host (Carvalho et al., 2010; Franzin et al., 2017). Although the reaction of neutrophils upon larval maturation to nymphs presents with conflicting results (Latif et al., 1991; Franzin et al., 2017), the lack of differential levels of this granulocyte seen in the larvae and adult life stage may support the hypothesis that this cell subtype remains unchanged across the tick life cycle.

#### Basophils at the site of tick attachment

The fluctuation in the number of basophils between tick-resistant and tick-susceptible cattle breeds infested with either one- or multi-host ticks have been studied. Interestingly, Latif et al. (1991) again identified a potential variation in host immune responses to different multi-host tick species. At *R. appendiculatus* nymph attachment sites, significantly fewer basophils were present in more susceptible compared to resistant cattle within and between breeds. In the same study, no significant differences in the number of basophils were identified between tick-susceptible and tick-resistant cattle within the *B. t. indicus* breed at the sites of *A. variegatum* nymph attachment. Yet, cattle of intermediate resistance showed the highest numbers of basophils. This can be explained by the study layout as the cattle group of lower resistance had previous exposure to much lower *R. appendiculatus* numbers compared to *A. variegatum*. Therefore, animals could be presenting with a higher resistance level against the latter species and thus account for observed discrepancies.

**Figure 1:**
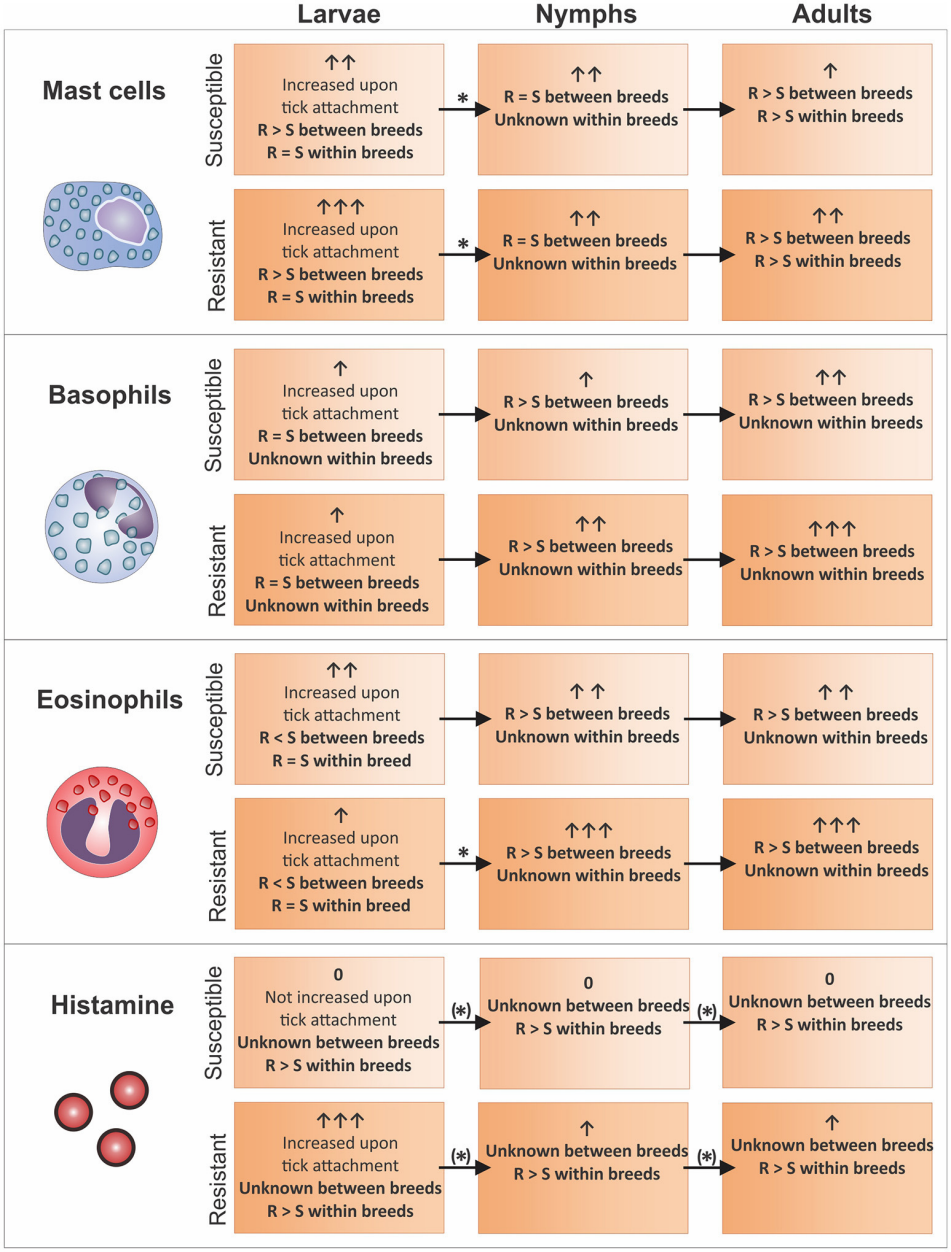
Mast cell, basophil, and eosinophil levels identified in the skin of cattle (sections on Basophils, Eosinophils and Mast Cells at the Site of Tick Attachment) with different tick-resistance status and their correlation with blood (section Translational Studies in the Blood of Tick-Infested Cattle) and skin (section Translational Studies and Metabolites in the Skin of Tick-Infested Cattle) histamine levels (based on literature investigating changes as result of *R. microplus* infestation). Granulocyte and histamine levels are indicated per tick life stage with arrows. An asterix indicates that a specific cell subtype dynamic between life stages was confirmed by literature as per relevant skin and blood section. Where dynamics were obtained from within breed comparisons the asterix was placed in brackets. Resistant and susceptible animals are abbreviated with “R” and “S”, respectively. Graphical representations correspond to the key of Figure 2.

In the one-host tick, *R. microplus*, basophil infiltration levels were also found to alter between cattle of varying resistance in response to tick infestation (Figure 1). Overall, basophil numbers at the site of adult tick attachment have been shown to be more abundant at tick attachment sites in resistant cattle than in their susceptible counterpart (Carvalho et al., 2010). Similarly, naturally infested cattle were found to have differing levels of basophils at the site of adult female *R. microplus* attachment, with tick counts negatively correlating with basophil counts (Marufu et al., 2014). The finding that basophil counts increase at the site of tick attachment in cattle was further corroborated by Franzin et al. (2017). This study showed that not only did both tick-resistant and tick-susceptible cattle recruit basophils at the site of *R. microplus* infestation, but also that upon maturation of *R. microplus* larvae to their nymph life stage, significantly more basophils were found to be present in resistant compared to susceptible hosts.

In summary, upon tick attachment basophil levels seem to increase in all cattle. The rate and level of increase is however dependent on the number of previous tick infestations and the level of tick resistance in the respective cattle breed. An increase in the number of infestations of a less susceptible breed to multi-host adult tick species not only showed an association with increased time taken to recruit basophils, but also increased number of basophils compared to previous tick infestations (Allen et al., 1977; Brown et al., 1984; Walker and Fletcher, 1986). Increased levels of this cell subtype in the nymph and adult life stages were found to be higher in resistant animals, with no difference identified between cattle breeds infested with tick larvae (Latif et al., 1991; Carvalho et al., 2010; Marufu et al., 2014; Franzin et al., 2017). These results suggest that it is not necessarily the difference in immune response pathway between cattle breeds that play a part in resistance but rather the level and reaction time of such immune responses.

#### Eosinophils at the site of tick attachment

##### Between breed comparisons of eosinophil levels

Altered patterns of eosinophil regulation were identified across different tick life stages, and will hence be discussed accordingly. Three conclusions can be drawn regarding the attachment of larvae on cattle. Firstly, upon tick larvae attachment, there was an overall increase in the number of eosinophils at the site of tick attachment in all cattle breeds. Susceptible cattle did, however, display a higher influx of eosinophils compared to their tick-resistant counterparts (Figure 1; Moorhouse and Tatchell, 1969; Piper et al., 2010; Franzin et al., 2017). Secondly, the infestation history of the host does not seem to play an important role regarding the levels of eosinophils at the larval life stage. This is supported by the observation that a higher influx of eosinophils to the attachment site occurs in susceptible breeds in naïve cattle as well as in cattle that have been repeatedly infested (Piper et al., 2010; Franzin et al., 2017). Lastly, differences are observed amongst larval infestation using different tick species. In the case of Moorhouse and Tatchell (1969) it was found that hours after the attachment of *R. microplus* larvae to cattle (with previous tick exposure), susceptible cattle presented with a greater number of eosinophils. In contrast, no difference in the influx of eosinophils was observed between resistant and susceptible cattle breeds in response to infestation with the multi-host tick, *Haemaphysalis longicornis*.

With regards to nymph infestation, Franzin et al. (2017) showed that a reversal of the larval eosinophil response is observed, where upon maturation of tick larvae to nymphs, a greater number of eosinophils occur in resistant breeds (Figure 1). The same trend seen during the nymph life stage continues into the adult life stage (Figure 1). This has been confirmed in studies using Shorthorn-Zebu vs. Shorthorn (Riek, 1962) and Nelore vs. Holstein-Friesian (Carvalho et al., 2010) cattle infested with adult *R. microplus*. One study did, however, not confirm this observation. Marufu et al. (2014) identified that the more susceptible Bonsmara (*B. t. afrikanus*) cattle displayed higher eosinophil levels compared to that of the resistant Nguni (*B. t. indicus*) cattle breed. To date, it is unknown what the cause of this discrepancy could be.

##### Within breed comparisons of eosinophil levels

In contrast to studies between cattle breeds, no difference in eosinophil levels at the tick larval life stage was found between animals of the same breed (Figure 1). Schleger et al. (1976) showed that in *B. t. taurus* infested with *R. microplus* larvae, similar numbers of eosinophils were present between animals of varying resistance. However, eosinophils were more localized to the site of tick attachment in more resistant cattle (Schleger et al., 1976). This is in contrast to what is seen in blood, where eosinophil levels are significantly higher in susceptible cattle, even under conditions of natural infestation (Rechav et al., 1990).

Regarding nymph infestation, differences in the response were observed with regards to what tick species the cattle were infested with. In a study by Latif et al. (1991) Zebu cattle infested with *A. variegatum* have a greater influx of eosinophils as opposed to cattle infested with *R. appendiculatus*. However, upon conducting intra-breed comparisons, Zebu cattle that displayed resistance to *R. appendiculatus* had lower eosinophil levels while no difference in eosinophil levels could be detected in *A. variegatum* susceptible or resistant animals. The latter observation could, however, be due to the presence of higher *A. variegatum* tick numbers compared to *R. appendiculatus* before the commencement of this study. As such, the tick species effect on eosinophil biology remains to be validated.

Studies focusing on the effects of multiple infestations (independent of the host-resistance status), indicated that eosinophil and degranulation levels progressively increase with the number of infestations with *Hyalomma anatolicum anatolicum* (Gill, 1986) and *R. appendiculatus* (Walker and Fletcher, 1986) adults. In contrast, Allen et al. (1977) showed that all *B. t. taurus* cattle infested with adult *Ixodes holocyclus* had increased eosinophil levels, irrespective of whether the animals were previously infested or not. However, the latter study should be confirmed due to the low numbers of biological repeats per cattle group and low tick numbers used.

Discrepancies observed for within a single breed to between different cattle breeds infested with tick larvae could indicate that eosinophils play different roles in the resistance mechanism in genetically more resistant breeds compared to acquired resistance within breeds. Since studies looking at the changes in eosinophil levels within breeds have mainly focused on *B. t. taurus* cattle, more studies should investigate changes in *B. t. indicus* animals of various resistance.

#### Mast cells at the site of tick attachment

Differences in mast cell numbers have been related to the tick life stage and differences have furthermore been found when comparing results from within and between breed studies. Upon *R. microplus* larvae attachment, there is an increase in the number of mast cells at the site of tick attachment in all cattle (Figure 1). This increase is intensified in more tick-resistant cattle (*B. t. indicus*) when compared to more susceptible animals (*B. t. indicus*) (Franzin et al., 2017) which was not seen in a study investigating effects within a *B. t. taurus* breed (Schleger et al., 1976; Figure 1).

Yet, upon maturation to nymphs, the number of mast cells are similar for both resistant and susceptible animals while a significant decrease in the number of mast cells in more resistant hosts is seen in cattle infested with nymphs compared to larvae (Figure 1; Franzin et al., 2017). Similarly, no significant changes in the number of mast cells in the skin of resistant and more susceptible animals infested with *R. appendiculatus* or *A. variegatum* nymphs was found between and within cattle breeds except for a suggested decrease of cells in less resistant animals within the Zebu breed (Latif et al., 1991).

When ticks reached the adult life stage, a greater number of mast cells at the site of tick attachment in more resistant cattle was observed in all studies (Figure 1; Engracia Filho et al., 2006; Veríssimo et al., 2008; Marufu et al., 2014). In a study by Engracia Filho *et al*. (2006), Gyr x Holstein cattle were grouped into resistant and susceptible groups based on previous infestations. Upon adult attachment of *R. microplus* it was shown that the number of mast cells in the more resistant group was greater than in the more susceptible group (Engracia Filho et al., 2006). Furthermore, this was confirmed in a similar study using a wider range of cattle breeds and resistance groups including Nelore, Holstein-Friesian, Brown, Gyr and crossbred animals which showed that in the upper dermis of *R. microplus* adult infested cattle skin there was a negative correlation between the number of ticks on the animals and the number of mast cells present (Veríssimo et al., 2008). Both tick-susceptible and more tick-resistant cattle skin naturally infested with *R. microplus* adults also showed a negative relationship between tick counts and mast cell numbers (Marufu et al., 2014).

In addition, investigation of the skin of *B. t. taurus* cattle infested with adult *H. a. anatolicum* suggested that irrespective of previous exposure to ticks, there is a negative correlation between the number of mast cells in the skin of cattle and the number of ticks attached to these animals (Gill, 1986). An increased number of mast cells was found at the tick bite lesion of tertiary as compared to primary infested animals. Additionally, degranulation of mast cells was seen in tertiary infested animals as opposed to naïve cattle. Allen et al. (1977) showed that in the case of adult *I. holocyclus* attachment on European cattle breeds, the number of mast cells in the skin increased upon tick attachment irrespective of previous exposure. It was also shown that mast cell infiltration and mast cell degranulation increased in previously exposed cattle as opposed to naïve cattle (Allen et al., 1977). In contrast, to the above results, *B. t. taurus* cattle infested multiple times with adult *R. appendiculatus* showed a decrease of mast cells at the tick attachment site (Walker and Fletcher, 1986).

In summary, as for the dynamic of the eosinophil cell subtype, differences in results were seen within and between cattle breeds. Mast cells were found to be at similar levels in susceptible and resistant cattle within a cattle breed. While between breeds, resistant cattle showed higher mast cell levels for the larval life stage (Schleger et al., 1976; Franzin et al., 2017). Similar results were obtained for studies investigating tick nymph and adult life stages between breeds.

## Dynamics of granulocytes and histamine and their suggested involvement in the tick-resistance mechanism over the tick lifecycle

**Figure 2:**
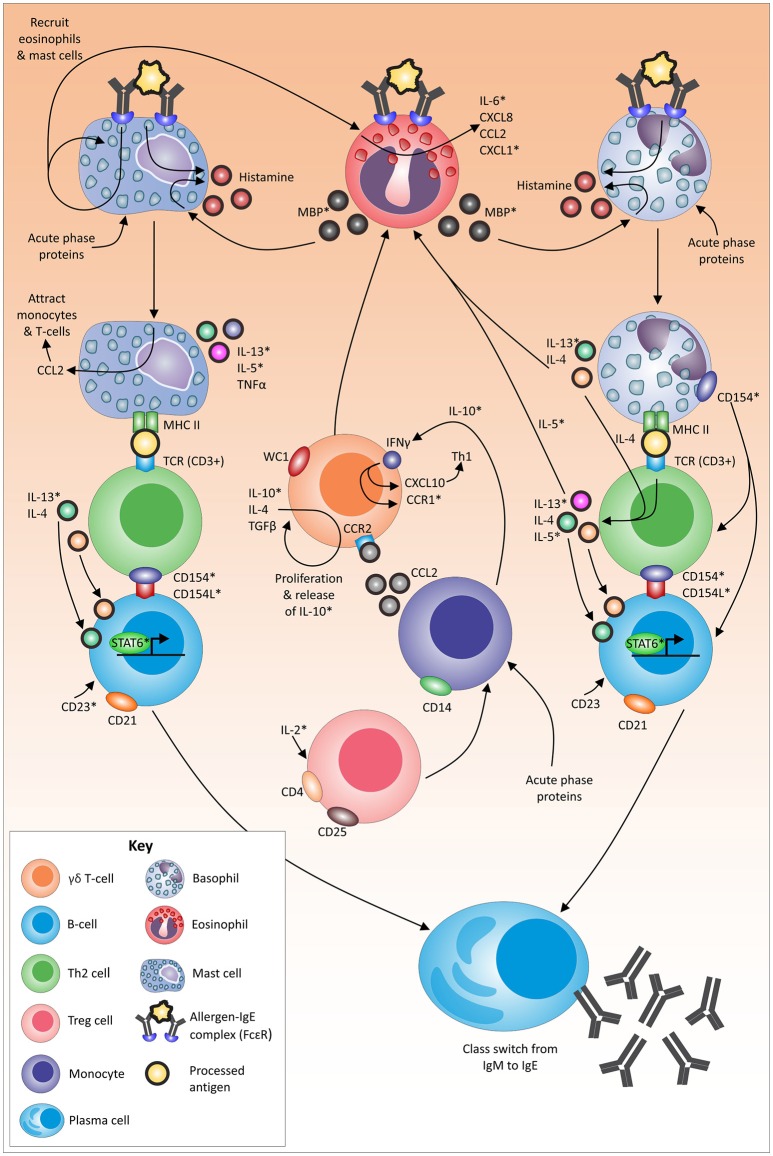
Proposed mechanism, role players and associated pathways in the tick-resistance mechanism. An asterix (*) indicates immune molecules that have not yet been identified to be linked to tick resistance/susceptibility in cattle (based on chosen exclusion criteria indicated in text). Molecules without an asterix have been linked to tick resistance/susceptibility in literature (as per relevant skin and blood section). Arrows indicate a direct or indirect link between molecules.

Changes in histamine and cell infiltration patterns over the life cycle of *R. microplus* stress the importance of taking the dynamics of cellular changes in response to the maturing tick into account when planning a study (Figure 1). In the case of histamine, it is increased in the tick larval life stage (in resistant animals) pointing toward it acting as an effector molecule within cattle breeds. In addition, we hypothesize that histamine is increased in resistant animals throughout all tick life stages based on four observations. Firstly, a study comparing susceptible and intermediate-resistant cattle identified higher blood histamine levels throughout the tick life cycle for the latter group within a single cattle breed (Riek, 1962). Secondly, higher histamine levels were found at the site of larval attachment of more resistant cattle within the same breed (Willadsen et al., 1979; Schleger et al., 1981). Thirdly, resistant breeds have equal or higher basophil (Carvalho et al., 2010; Marufu et al., 2014; Franzin et al., 2017); and mast cell (Schleger et al., 1976; Engracia Filho et al., 2006; Marufu et al., 2014; Franzin et al., 2017;) levels throughout all life stages compared to susceptible breeds. Lastly, as histamine can be released from mast cells as well as basophils via an IgE and/or eosinophil-dependent mechanism, the presence of these cells correlate with the increase in histamine observed (Ishizaka et al., 1972; Zheutlin et al., 1984; Janeway et al., 2001; Galli et al., 2005; Stone et al., 2010) (Figure 2).

### Comparison of findings obtained within a single cattle breed

Although no differences were observed for mast cell and eosinophil levels at the larval life stage, histamine levels were found to be higher in resistant compared to susceptible animals (Figure 1) (Riek, 1962; Willadsen et al., 1979; Schleger et al., 1981). Potentially, histamine is thus released via basophils, however, no study up to date has extensively investigated this cell subtype within a single breed. It should be noted that Riek (1962) only investigated the dynamics of this compound during the larval life stage in a limited study using only one highly resistant animal. Higher histamine levels upon nymph and adult tick attachment in blood were also identified when comparing medium resistant to susceptible cattle (Figure 1; Riek, 1962). If histamine levels were linked to tick numbers as result of tissue damage, it would be expected that higher histamine levels are present in more susceptible animals which is not the case. This indicates that histamine could be involved in the tick-resistance mechanism within cattle breeds. Since not enough data regarding the number of granulocytes are available to date for the nymph and adult life stages, it cannot be hypothesized by which cell subtype histamine may be released. However, increased mast cell numbers in resistant compared to susceptible animals at the adult life stage could indicate a delay in mast cell dependent release of histamine as seen for inter-breed comparisons at the larval life stage (section Comparison of Findings Obtained between Different Cattle Breeds).

### Comparison of findings obtained between different cattle breeds

Although mast cells increased upon larvae attachment for all cattle breeds evaluated, this cell subtype was found to be more abundant in resistant breeds (Figure 1) (Franzin et al., 2017). This, together with the equal levels of basophils in both resistant and susceptible cattle at the tick larval life stage (Figure 1; Franzin et al., 2017), indicates that histamine might be increased as a result of mast cell degranulation and contributes to the first line of tick defense. The higher eosinophil levels in susceptible compared to resistant cattle breeds at the larval life stage (Figure 1; Moorhouse and Tatchell, 1969; Piper et al., 2010; Franzin et al., 2017) furthermore suggests that the immune response in susceptible cattle increases histamine levels via an eosinophil-dependent mechanism. This mechanism might be less efficient and slower in susceptible cattle due to its involvement in late-phase reactions (Piliponsky et al., 2001). Mast cell levels in the nymph life stage were found to be present at similar levels in resistant and susceptible breeds (Latif et al., 1991; Franzin et al., 2017), while a relatively higher number of mast cells was seen in more tick-resistant cattle in the adult tick life stage (Figure 1). This could be a result of a decrease of this cell subtype in susceptible and not an increase in resistant animals. The apparent decline in the number of mast cells that was observed in the more resistant cattle breed from the larvae to the nymph life stage may thus be delayed and occurring in the more susceptible cattle at the adult life stage. Since the tick-resistance mechanism in *B. t. indicus* animals results in a generally faster response to tick infestation compared to *B. t. taurus* cattle (Riek, 1962; Wagland, 1978, 1980; Rechav et al., 1990), resistance within susceptible cattle breeds might be achieved through a delayed histamine release via basophils. This mechanism is suggested to occur at the nymph life stage in resistant cattle breeds (Figure 1). To specifically elucidate this resistance mechanism in depth, especially granulocyte levels for animals within a breed, presenting with varying tick-resistance phenotypes, need to be determined throughout the tick life cycle. Additionally, investigation of histamine within and between cattle breeds at all tick life stages are essential.

## Future directions: potential drivers involved in tick resistance

This integrative discussion will give an evaluation of key role players investigated up to date to establish a global view of components potentially involved (directly or indirectly) in the tick-resistance mechanism. It must be kept in mind that some observed immune responses may be a by-product of the effector pathway/molecule or a response to tick infestation without any involvement in the actual resistance mechanism. For example, gene expression results and actual dynamics occurring on protein level often do not correlate due to post-transcriptional, post-translational and degradation regulation (Vogel and Marcotte, 2012). Therefore, results from studies employing gene expression analysis were only included as part of this discussion if their findings have been validated by a second study. Furthermore, since gamma globulin levels, apart from IgE, were generally increased in susceptible cattle, this could be linked to elevated tick numbers. Even though antibody specificity could be a contributing factor, these molecules were thus not included as key role players in this section as evidence remains non-conclusive. Lastly, due to a constant dynamic of immune molecules, identified markers on translational and cellular levels were included if a significant difference (irrespective of the direction) was seen between animals with more and less resistance status. Figure 2 summarizes potential role players and their possible interactions driving resistance, based on findings up to date with the discussion providing an integrative explanation of identified immune marker interactions of the respective components supported by literature. Indicated with an asterix (^*^) in the text below and in Figure 2, are components that have not yet been identified to be linked to tick resistance/susceptibility in cattle.

During the process of tick attachment, the skin of the host is damaged/pierced, and tick saliva is exposed to sentinel cells, such as granulocytes. Several acute-phase proteins have been shown to be involved at the site of tick attachment (Carvalho et al., 2008). These proteins can be linked to granulocyte (Quaye, 2008; Stone et al., 2010; Eklund et al., 2012) and monocyte (Hochepied et al., 2003) recruitment and/or activation. Furthermore, tick secreted allergens can cross-link to IgE (Galli and Tsai, 2012) and binding of IgE to its high-affinity receptor (FcεRI) on dermal mast cells (and basophils) has been shown to lead to the release of inflammatory mediators (Stone et al., 2010) such as histamine (Galli et al., 2005).

Following activation, mast cells readily secrete IL-5^*^, IL-13^*^, and TNFα (Janeway et al., 2001; Stone et al., 2010). In addition, IgE binding leads to the enhancement of CCL2 (monocyte chemoattractant protein 1) transcription that promotes the migration of monocytes (Oliveira and Lukacs, 2001) and T-cells (Oliveira and Lukacs, 2003) to amplify the local inflammatory reaction. Lastly, due to the antigen presenting nature of mast cells, following the uptake of the IgE-antigen complex, the allergen is presented on mast cells on MHC II, which in turn interacts directly with T-cell receptors (containing CD3) and induces antigen-specific clonal expansion of T-cell populations (Mekori and Metcalfe, 1999; Henz et al., 2001).

Basophils also function as antigen presenting cells in response to certain allergens (Sokol et al., 2008, 2009). The binding of the IgE-allergen complex to FcεRI on basophils activates several pathways in the cell resulting in the release of histamine (Ishizaka et al., 1972) and the expression of IL-4 and IL-13^*^ (Stone et al., 2010). These cytokines are important for the promotion of eosinophil trafficking (Stone et al., 2010) and are also secreted by Th2 cells in response to the presentation of allergen via MHC II and IL-4 production (Perrigoue et al., 2009; Yoshimoto et al., 2009) Activated Th2 cells also secrete cytokines (e.g., IL-5^*^) which increases eosinophil production (Janeway et al., 2001).

Antigen presentation to Th2 lymphocytes by mast cells and/or basophils, provide two essential signals for isotype switching. The first signal is IL-4 and/or IL-13^*^ which bind to the respective receptors on B-cells and activate transcription at the IgE isotype-specific site via STAT6 (Stone et al., 2010). The second signal involves the binding of CD40L (CD154L^*^) to the relevant T-cell receptors, which in turn activates DNA switch recombination (Stone et al., 2010). Basophils express high levels of CD154^*^ after activation and have been suggested to play a role in polyclonal amplification of IgE production and in the differentiation of Th2 cells (Stone et al., 2010). In addition, the binding of CD23^*^ to CD21+ B-cells may participate in the control of IgE production (Aubry et al., 1992).

Histamine can also be released from mast cells and basophils via an IgE-independent mechanism (Siraganian and Hook, 1976; Piliponsky et al., 2001) utilizing the major basic protein^*^ released from eosinophils (Zheutlin et al., 1984; Janeway et al., 2001). The binding of the allergen-IgE complex to mast cells is suggested to drive the recruitment and activation of additional mast cells and eosinophils (Wong et al., 2009). Mast cells can also induce the release of IL-6^*^, CXCL8, CCL2 and CXCL1^*^ by eosinophils (Wong et al., 2009).

The development of eosinophilic allergic inflammation and the initiation of Th2-responses is regulated by a T-cell subtype (Zuany-Amorim et al., 1998). Regulatory T-cells are generally known for their ability to suppress putative deleterious activities of Th cells (Corthay, 2009), with IL-2^*^ playing an important role in the survival and proliferation of CD4+CD25+ regulatory T-cells (Létourneau et al., 2009). The exact role of CD4+CD25+ regulatory T-cells in bovines is, however, unknown. It has been proposed that this cell population is neither anergic nor suppressive in cattle, and that their function(s) can to be linked to γδ T-cells (WC1.1+, WC1.2+) (Hoek et al., 2009). Together with the latter cell type, CD14+ monocytes have been linked to immune suppression in ruminants (Hoek et al., 2009). In addition, γδ T-cells bearing the lineage marker WC1 are associated with the production of the proinflammatory cytokine IFNγ (Rogers et al., 2005), that furthers the action of CXCL10 (an INFγ inducible protein, which recruits activated Th1 cells to the site of inflammation) (Dufour et al., 2002). Interferon gamma (Zella et al., 1998) as well as other cytokines such as IL-10^*^ on monocytes (Loetscher et al., 1996; Sozzani et al., 1998) can mediate upregulation of CCR1^*^ expression. The release of IL-10^*^, IL-4 and TGFβ can result in the proliferation of subsets of γδ T-cells (Guzman et al., 2014). The accumulation of γδ T-lymphocytes during allergic inflammation in turn is orchestrated by the CCR2/CCL2 pathway (Costa et al., 2009; de Oliveira Henriques and Penido, 2012).

Several immune markers associated with the above pathways, such as histamine, have been identified in section Blood and Skin Tissue (Figure 2). To date, histamine levels in the blood and skin were found to be increased in resistant cattle, while little variation was seen in susceptible animals (Riek, 1962; Willadsen et al., 1979; Schleger et al., 1981). Degranulation of both mast cells (Riley, 1953; Mota et al., 1954) and basophils (Pruzansky and Patterson, 1970) is followed by the subsequent release of histamine in an immediate hypersensitivity reaction (Ishizaka et al., 1970, 1972). This reaction is well known to be linked to more frequent and intense grooming (O'Mahony et al., 2011), which was identified to play a role in tick-resistance (Riek, 1956; Snowball, 1956). Future studies are now required to elucidate these predicted pathways in depth, focusing on likely markers not yet investigated (^*^) and molecular mechanisms/molecules that have resulted in contradicting findings up to date. Since opposing findings could be linked to varying study time points, a dynamic investigation of all potential role players would be valuable and could lead to a better-defined picture of occurrences.

## Critical evaluation and concluding remarks

To date, few immune markers have been investigated with sufficient depth to obtain a picture of events involved in the cattle tick-resistance mechanism. Especially large-scale transcriptome studies have identified various components with hardly any overlap of results between studies. One reason for this is the differences in experimental designs which can drastically influence the occurrence of immune events depicted at the chosen time point corresponding to a specific tick life stage(s). In addition, potential markers or pathways should not only be studied on a molecular level but also on a cellular level. The clearest pictures, as obtained for histamine, granulocytes and gamma globulin levels, were identified using markers on both translational and cellular level. In this regard, some immune markers seem to differ for the resistance mechanism within a host breed compared to between breeds as well as between tick life stages (see section Dynamics of Granulocytes and Histamine and their Suggested Involvement in the Tick-Resistance Mechanism Over the Tick Lifecycle and Figure 1). It should be noted that even though the final effector molecules might be the same, different pathways are possibly involved in establishing this mechanism (see section Future Directions: Potential Drivers Involved in Tick Resistance). Several experimental factors should thus be considered when comparing experiments as some of these parameters can potentially skew results and lead to contradicting findings if not addressed correctly (Table 1). This includes important aspects and potential solutions regarding (1) factors relating to the selection and treatment of host animals, (2) factors relating to infestation and sampling protocols, (3) selection of immune markers and (4) data analysis and interpretation.

**Table 1:** Problem identification and potential solutions for studies evaluating the interplay of cattle immune responses to tick infestation.

**Problem statement/explanation**	**Possible solutions and future guidelines**
**Factors relating to the selection and treatment of host animals**	
Extrapolation of findings between different host species (e.g., rodents and bovines).	A rodent model can be used to provide hypotheses, as numerous validated immune markers are available for murine models. However, a significant amount of results cannot be directly extrapolated from murine to bovine hosts and should therefore be confirmed in the appropriate host species to validate an immune response.
Not considering intra-breed differences (range of tick-resistance status between individual hosts within a breed).	Cattle should be sourced from registered breeders to limit genetic differences to a minimum. The resistance status of each animal needs to be taken into account when analyzing data and confirmed prior to the start of a study.
Lack of patient history of experimental animals.	Information on animal source, age and previous exposure(s) to ticks should be provided.
Reporting prophylactic treatments of host animals upon arrival and health status throughout study.	Upon arrival, all treatments should be reported with special emphasis on acaricide treatments and prophylactic treatments (antibiotics for infections, deworming strategies), as all of these influence immunity. General health parameters such as weight, temperature and hematocrits need to be reported as bovine studies are rarely conducted under biosafety level standards.
**Factors relating to infestation and sampling protocols**	
Comparison of immune responses between animals with different tick attachment efficiencies and thus tick numbers at the respective sampling time points.	Comparing immune responses of susceptible and resistant hosts infested with the same number of ticks (especially at nymph and adult life stages) would allow for a better understanding of which mechanism is at play at which life stage.
Protocol for tick infestation and evaluation of specific tick life stage(s).	Biological question should take into account infestation protocol, as multiple infestations results in numerous life stages being present on a single animal that will bias detection of a life stage specific response.
Focus on between animal comparisons at specific time points instead of immune dynamics.	Focus should be placed on the progression of immune responses within breeds and then determine between breed differences.
Choice of biological sample for analyses.	To date, skin and blood have been studied extensively. Insights from secondary lymphoid organs are in dire need to fully understand tick-mediated immune suppression as well as factors underlying tick resistance.
**Selection of immune markers**	
Translation of findings to all cellular levels.	Gene expression profiling studies should be validated using protein and/or cellular markers. Genotyping studies should take into account cellular immune markers to link genotypes and phenotypes.
Most immune markers are not confirmed as cross-reactive to specific cell subpopulations in bovines or are lacking.	Immune markers must be confirmed to be cross-reactive to a specific cell subpopulation.
**Data analysis and interpretation**	
Comparison between studies investigating different tick species (e.g. one-host vs. multi-host ticks).	Transcriptome analysis across ixodid tick species support differences in proteins being present in a specific species. Therefore, cross-species comparisons should be carefully considered.
Comparison between studies investigating different tick life stages.	A unique set of proteins/molecules can be secreted by each life stage of a tick species, with a unique subset of immune cells affected/targeted (e.g., see section on granulocytes).

To date, the only partially clear picture of immune events involved in the tick-resistance mechanism involves histamine and associated cell subtypes and molecules (Figures 1, 2). This pathway, with its effector molecules and modes of action, still requires additional in-depth investigation before focus is placed on the identification of other contributing mechanisms. The identified cell dynamics between tick life stages furthermore suggest that future studies should concentrate on the dynamics of immune responses at various time points over the complete tick life cycle. This would reduce between study variations in addition to obtaining a temporal overview of events. In general, it is advisable that experiments are standardized as much as possible and more focus should lie on an in-depth investigation of markers/pathways across genetic, translational and cellular levels to successfully validate a response. Only then can one attempt to consolidate all information into a feasible blue print for the identification of major drivers underlying the bovine immune mechanism driving tick-resistance and subsequent postulation of viable and effective tick control strategies.

## Author contributions

LR, SR, CM-O: conceptualized manuscript focus, critically revised draft manuscript, approved final manuscript; LR, SR: accumulated data, designed Figures and Tables, wrote first manuscript draft.

### Conflict of interest statement

The authors declare that the research was conducted in the absence of any commercial or financial relationships that could be construed as a potential conflict of interest.

## Supplementary material

Supplementary Table 1 Summary of studies investigating tick resistance/ susceptibility categorized according to tissue of study.
